# Impact of comorbidities in patients with erosive hand osteoarthritis (EHOA), a monocentric study

**DOI:** 10.3389/fragi.2026.1748066

**Published:** 2026-02-12

**Authors:** S. Bindoli, G. Cozzi, C. Benvoluti, M. Lorenzin, S. Vio, P. Sfriso, M. Favero, R. Ramonda

**Affiliations:** 1 Rheumatology Unit, Azienda‐Ospedale Università di Padova, Padova, Italy; 2 Department of Medicine (DIMED), University of Padova, Padova, Italy; 3 Radiology Unit, Padova University Hospital, Padova, Italy; 4 Internal Medicine 1, AULSS2 Marca Trevigiana, Ca’ Foncello Hospital, Treviso, Italy; 5 Department of Surgery, Oncology, Gastroenterology (DiSCOG), University of Padova, Padova, Italy

**Keywords:** chronic pain, comorbidities, metabolic syndrome, osteoarthritis, osteoporosis

## Abstract

**Objective:**

Erosive hand osteoarthritis (EHOA) is a severe and rapidly progressing form of osteoarthritis that has been linked to systemic comorbidities (i.e., metabolic bone and cardiovascular diseases). The object of this study is to retrospectively evaluate the impact of comorbidities (i.e., osteoporosis, diabetes and overweight) on the clinical course and radiographic findings in a cohort of EHOA patients.

**Design:**

This is a retrospective cross-sectional study. Patients underwent clinical assessments and completed the VAS scale, the AUSCAN and DREISER questionnaires. Metabolic, cardiovascular, and bone health data were collected. Radiographic features—osteophytes, joint space narrowing, malalignment, erosions, sclerosis, and subchondral cysts—were evaluated using the Altman system. Comorbidities were assessed using the Charlson Comorbidity Index, whereas metabolic syndrome, diabetes, and osteoporosis were defined according to the ATP III and WHO criteria. Statistical analysis was conducted via Spearman’s correlation, using GraphPad Prism 9.1.0, with significance set at p < 0.05.

**Results:**

Eighty-seven patients (88.5% female, mean age 63.17 ± 8.85) were included. Among comorbidities, 76.8% had at least one risk factor; BMI correlated with joint space narrowing (r = 0.22, p = 0.04). Severity of femoral and lumbar osteoporosis correlated with AUSCAN and DREISER scores; FRAX scores significantly correlated with several radiographic features of EHOA; VAS correlated with swollen and painful joint count, with AUSCAN and DREISER scores and with osteophytosis. Disease duration correlated with overall radiographic damage.

**Conclusion:**

Cardiovascular and metabolic bone comorbidities, especially overweight and osteoporosis, appear to be associated with higher pain burden, functional impairment, and greater structural damage in EHOA patients.

## 1 Introduction

**FIGURE 1:**
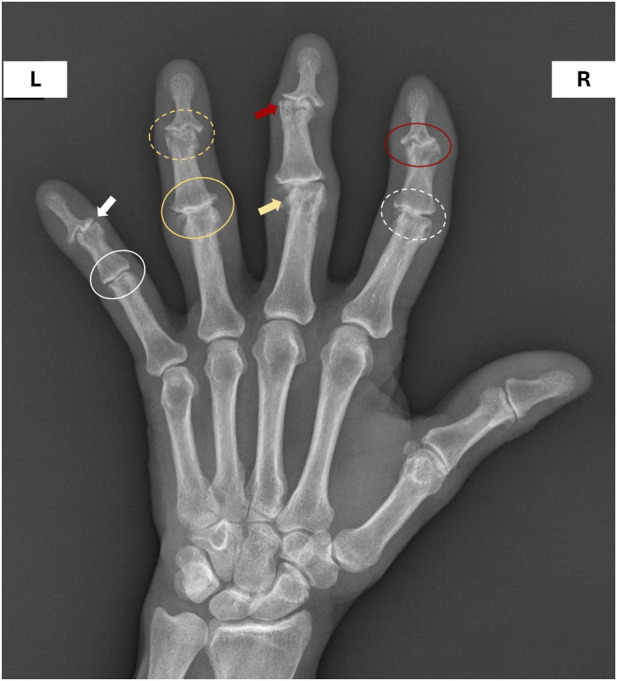
Representative radiograph of erosive hand osteoarthritis showing radiographic features scored according to the Altman system. Example of a left hand X-ray from a patient with erosive hand osteoarthritis (EHOA), illustrating the main structural changes assessed by the Altman radiographic scoring system, including osteophytes, joint space narrowing, malalignment, erosions, subchondral sclerosis, and subchondral cysts. Legend: R = right, L = left; side white circle: grade 1 joint space narrowing; yellow circle: grade 3 joint space narrowing; white dashed circle: grade 1 marginal osteophyte; yellow dashed circle: grade 3 marginal osteophyte; white arrow: subchondral sclerosis; red arrow: subchondral cyst; yellow arrow: malalignment; red circle: erosion.

Erosive hand osteoarthritis (EHOA) is a subset of hand osteoarthritis (HOA) that is defined by the presence of at least one erosion in interphalangeal (IP) joints on plain radiographs and occurs primarily in postmenopausal women (Punzi et al., 2004; Favero et al., 2022). The most affected joints in EHOA patients are the proximal (PIP) and distal interphalangeal (DIP) joints, typically sparing the metacarpophalangeal joints. A distinguishing feature of EHOA is the simultaneous involvement of multiple joints, unlike in typical HOA, which generally affects joints progressively and individually (Favero et al., 2022). The second and third fingers are most affected, often symmetrically, followed by the fourth and fifth fingers (Figure 1). The overall prevalence of EHOA is currently estimated at about 2.8%, though it may reach 10.2% among individuals with symptomatic OA (Kwok et al., 2011).

According to European Alliance of Associations for Rheumatology (EULAR), EHOA differs from classic HOA presents distinct features and characteristics from the classical HOA, and namely its acute onset and peculiar radiographic pattern characterised by central erosions in the subchondral bone, accompanied by cortical bone destruction and subsequent remodelling, which can lead to ankylosis of the affected joints (Punzi et al., 2004; Zhang et al., 2007; Marshall et al., 2018). Furthermore, EHOA is also characterised by clinical and radiographic signs of inflammation. Several studies have demonstrated inflammation of the synovial membrane in the interphalangeal joints using ultrasound and MRI (Haugen et al., 2016; Iagnocco et al., 2005). Additionally, biomarker analyses have detected elevated blood levels of pro-inflammatory cytokines (Beekhuizen et al., 2013; Punzi et al., 2012) and high-sensitivity C-reactive protein (hs-CRP) levels (Smith et al., 2012; Punzi et al., 2005). Notably, synovial inflammation in EHOA correlates closely with patients’ symptoms and the development of new bone erosions (Kortekaas et al., 2016; Mancarella et al., 2015).

Several metabolic and cardiovascular diseases have been associated with HOA, including osteoporosis (OP) (Kasher et al., 2020) and obesity (Kluzek et al., 2015; Brogren et al., 2024). However, the evidence linking metabolic syndrome (MetS) to OA remains inconsistent (Strand et al., 2018). While OA in general does not appear to be strongly associated with MetS, certain phenotypes, such as nodal OA and EHOA may have shown a stronger connection, suggesting a potentially distinct pathophysiological mechanism of this latter form (Brogren et al., 2024). Indeed, low-grade chronic inflammation, combined with altered biomechanical loading, appears to play a critical role in the pathogenesis of EHOA influencing both clinical presentation and disease progression.

Our study endeavoured to ascertain the specific impact of cardiovascular and metabolic bone comorbidities on the clinical course and radiographic findings—osteophytes, joint space narrowing, malalignment, erosions, sclerosis, and subchondral cysts—in a cohort of EHOA patients. Additionally, we aimed to investigate the relationship between cardiovascular comorbidities and the presence of osteoporosis using clinical and serological indices of joint disease activity, including pain Visual Analogue Scale (VAS), disease activity questionnaires, namely the Australian/Canadian Osteoarthritis Hand Index (AUSCAN) (Bellamy et al., 2002) and Dreiser Functional Index (DREISER) (Dreiser et al., 1995), tender (TJ) and swollen joints (SJ) count, along with inflammatory biomarkers.

## 2 Methods

In this retrospective cross-sectional study were included 87 patients (female 88.5%, mean age 63.17 ± 8.85 years) with a confirmed diagnosis of EHOA which were followed at the Hand Osteoarthritis Clinic of Padova University Hospital, Italy, between January 2015 and February 2025. For the present study, all clinical, laboratory, and radiographic variables were extracted from the index visit used as the cross-sectional timepoint. Inclusion criterion required the presence of at least one central erosion in a PIP or DIP joint on hand plain radiographs. Patients with erosions involving metacarpophalangeal joints were excluded from the study. All participants provided written informed consent. The study protocol was approved by the local Ethics Committee (approval number 446/AO/23, dated 12 February 2024) and the study was conducted in accordance with the principles of the Declaration of Helsinki.

Each patient underwent a comprehensive clinical evaluation, including a detailed joint examination conducted by an experienced rheumatologist to assess SJ and TJ counts. Patients completed the following validated tools/questionnaires for the assessment of hand osteoarthritis at a single timepoint visit:

- VAS for hand pain, scored on a scale from 0 (no pain) to 10 (worst possible pain);
- AUSCAN, which evaluates pain, stiffness, and physical function, with scores ranging from 0 (no impairment) to 4 (severe impairment);
- DREISER, which assesses functional ability in hand osteoarthritis using a 4-point scale (0 = no difficulty, 4 = extreme difficulty in performing daily activities).

The following demographic, clinical, and laboratory variables were collected: age, sex, age at disease onset, comorbidities, ongoing pharmacological treatments, erythrocyte sedimentation rate (ESR), and C-reactive protein (CRP) levels. We measured the following metabolic parameters: body mass index (BMI), blood pressure, and serum levels of uric acid, glucose, total cholesterol, high-density lipoprotein (HDL), low-density lipoprotein (LDL), and triglycerides. We also assessed the following variables of metabolic bone diseases: history of fragility fractures, family history of fractures, age of menopause, smoking status, bone mineral density (BMD) values, and serum and 24-h urinary levels of calcium and phosphate, parathyroid hormone (PTH), and 25-hydroxyvitamin D levels.

### 2.1 Radiographic evaluation

We employed the Altman scoring system (Altman and Gold, 2007) to assess radiographic features such as osteophytes, joint space narrowing, malalignment, central erosions, subchondral sclerosis, and cysts. Scoring was applied to specific joint sites as follows:

- Osteophytes and joint space narrowing were scored in the DIP, PIP, first interphalangeal (1^st^ IP), TMC, and trapezium-scaphoid (TS) joints, with a 0–3 scale for DIP, PIP, and TMC, and a binary 0/1 scale for 1^st^ IP and TS.
- Malalignment, central erosions, and subchondral sclerosis were scored as present or absent (0/1) in DIP, PIP, and TMC joints.
- Subchondral cysts were evaluated in PIP and TMC joints using a binary scale (0/1).

Altman scoring atlas was used as reference, and training sessions were conducted by an experienced Radiologist.

The inter-reader agreement in the radiographic scoring evaluation was found to be substantial for the assessment of osteophytes, with a weighted kappa coefficient of 0.72 (95% confidence interval: 0.67–0.78). Moderate agreement was observed for the following variables: malalignment (Cohen’s kappa = 0.60, 95% CI: 0.49–0.72), sclerosis (kappa = 0.60, 95% CI: 0.48–0.71), joint space narrowing (weighted kappa = 0.50, 95% CI: 0.43–0.58), and erosions (kappa = 0.55, 95% CI: 0.42–0.67). In contrast, the inter-reader agreement for the scoring of subchondral cysts was lower, with a Cohen’s kappa of 0.30 (95% CI: 0.18–0.42), reflecting a fair level of concordance between the two observers.

### 2.2 Comorbidity assessment

The Charlson Comorbidity Index (CCI) (Charlson et al., 1987) was employed to quantify the burden of comorbid conditions in the cohort. This validated index predicts long-term mortality risk by assigning weighted scores to various diseases, including cardiovascular diseases (myocardial infarction, heart failure), chronic kidney disease, chronic respiratory diseases, cancer, liver disease, diabetes, neurological disorders (stroke, dementia), peptic ulcer disease, and rheumatoid arthritis. Scores range from 1 to 6 based on disease severity, with metastatic cancer carrying the highest weight. The total CCI score is the sum of individual comorbidity scores, with higher totals indicating greater mortality risk. In this study, CCI was calculated retrospectively from patient medical histories.

### 2.3 Metabolic syndrome evaluation

Metabolic syndrome was defined according to the Adult Treatment Panel (ATP) III criteria of the National Cholesterol Education Program (NCEP) (Expert Panel on Detection et al., 2001). Diagnosis required at least three of the following: abdominal circumference >102 cm in men or >88 cm in women; triglycerides ≥150 mg/dL (or treatment); HDL cholesterol <40 mg/dL in men or <50 mg/dL in women (or treatment); blood pressure ≥130/85 mmHg (or treatment); fasting glucose ≥100 mg/dL or diagnosis of type 2 diabetes (or treatment).

### 2.4 Fracture risk assessment

Fracture risk was estimated using the Fracture Risk Assessment Tool (FRAX^®^), a widely validated algorithm (http://frax.shef.ac.uk/FRAX) (Kanis et al., 2007). The FRAX score computes the 10-year probability of both hip fractures and major osteoporotic fractures (clinical spine, hip, forearm, or humerus). The tool incorporates age, sex, and body mass index (BMI) together with seven easily obtainable dichotomous clinical risk factors: prior fragility fracture, parental history of hip fracture, current smoking, systemic glucocorticoid use, excess alcohol intake, rheumatoid arthritis, and other secondary causes of osteoporosis.

### 2.5 Statistical analysis

Data normality was checked using the Shapiro-Wilk test. Categorical variables were compared using Chi-square or Fisher’s exact tests, and continuous variables with Mann-Whitney U or unpaired t-tests. Correlations between continuous variables were assessed by Spearman’s or Pearson’s coefficients, focusing on moderate to strong associations. To investigate potential relationships between recorded metabolic bone comorbidities, particularly diabetes, OP, BMI, and joint damage, pain, and disability, multiple correlations among these variables were analysed using Spearman correlation which evaluated relationships between radiographic, clinical, and densitometric variables. The agreement of the various characteristics of the radiographic scoring between the two readers was evaluated using Cohen’s kappa for dichotomous variables such as erosions, malalignment, cysts, and sclerosis, and the weighted kappa test for ordinal variables such as osteophytes and joint space narrowing. The interpretation of the kappa or weighted kappa coefficients was as follows: <0.00: poor agreement; 0.00–0.20: slight agreement; 0.21–0.40: fair agreement; 0.41–0.60: moderate agreement; 0.61–0.80: good agreement; 0.81–1.00: very good agreement. Associations between clinical, metabolic, densitometric and radiographic variables were evaluated using Spearman’s rank correlation coefficient, and all reported associations represent bivariate correlations. Given the exploratory nature of this retrospective cross-sectional study and the number of tested associations, no formal correction for multiple testing was applied. Therefore, all results should be interpreted as hypothesis-generating.

Analyses were performed using GraphPad Prism 9.1.0; a p < 0.05 was considered significant.

## 3 Results

Eighty-seven EHOA patients [females 77 (88.5%), males 10 (11.5%)], mean age of 63.17 ± 8.85 years, were included. The clinical and demographic characteristics along with biological exams, including CRP, ESR, VAS scale AUSCAN/DREISER questionnaires scores are summarised in Table 1. Regarding the radiological assessment, bilateral hand X-rays were collected from 86 patients. For each patient, 22 joints were examined, with an overall 1.892 joints evaluated. Radiographic features of the study population are detailed in Supplementary Table S1. When assessing osteophytes in the DIP, PIP, and TMC joints, 23.54% had no osteophytes, while the remaining joints showed varying degrees, with a grade 3 achieved 25.61% overall, with DIP joints being more involved by the presence of grade 3 osteophytes (30.61%). The pattern of joint space narrowing was similar to that of osteophytes with only 10.08% of DIP, PIP, and TMC joints being unaffected, with grade 3 achieved in 26.23% of the joints overall. Grade 3 narrowing was most common in DIP joints (38.52%). Malalignment was present in 44.50% of DIP, PIP, and TMC joints, with the highest incidence in TMC joints (49.42%). Erosions were found in 40.49% of joints, most commonly in DIP joints (55.10%). Sclerosis was present in 45.85% of joints, more frequently in TMC joints (58.33%). Subchondral cysts were detected in 36.54% of joints, especially in DIP joints (46.06%). When analysing median total radiographic scores per patient, including osteophytes: 29.29 ± 11.90 (range: 0–58), joint space narrowing: 32.81 ± 9.38 (range: 0–58), malalignment: 8.00 ± 3.34 (range: 0–18), erosions: 7.28 ± 3.93 (range: 0–18), sclerosis: 8.23 ± 3.43 (range: 0–18), cysts: 6.57 ± 2.90 (range: 0–18). Cardiovascular and metabolic bone comorbidities exhibited by the patients are shown in Table 2. Overall, 75.86% of participants had at least one cardiovascular risk factor including hypercholesterolemia, hypertriglyceridemia, obesity, and arterial hypertension.

**TABLE 1:** Clinical, laboratory and demographic data of the patients included.

Demographic, clinical and laboratory baseline data	Value
Age (years), mean ± SD	63.17 ± 8.85
Female sex, n (%)	77 (88.5%)
Disease duration (years), mean ± SD	13.30 ± 8.83
Smoking status	
- Current smokers, n (%)	7 (8.1%)
- Former smokers, n (%)	21 (24.4%)
- Non-smokers, n (%)	58 (67.4%)
Number of tender joints (0–30), mean ± SD	5.39 ± 4.89
Number of swollen joints (0–30), mean ± SD	0.99 ± 1.56
ESR (mm/h), median (IQR)	12.5 (6.25–20.75)
CRP (mg/L), median (IQR)	2.9 (0.98–3.1)
VAS pain score (0–10), mean ± SD	5.58 ± 2.61
AUSCAN score (0–60), mean ± SD	29.92 ± 12.37
DREISER score (0–30), mean ± SD	10.36 ± 6.25
FRAX	Percentage (%)
<5%, n (%)	29 (49.2%)
5%–10%, n (%)	17 (28.8%)
>10%, n (%)	13 (22.0%)

AUSCAN, Australian Canadian osteoarthritis hand index; CRP, C-Reactive Protein; DRISER, dreiser osteoarthritis severity index; ESR, erythrocyte sedimentation rate; FRAX, fracture risk assessment tool; VAS, visual analogue scale.

**TABLE 2:** Cardiovascular comorbidities along with osteometabolic parameters of the patients included.

Assessed variables	Patients N (%)
Laboratory test (mg/dL)	
Total cholesterol >200, n (%)	42 (48.27%)
Total cholesterol >240, n (%)	10 (11.49%)
LDL cholesterol >100, n (%)	54 (62.07%)
LDL cholesterol >130, n (%)	29 (33.33%)
Triglycerides >150, n (%)	5 (5.75%)
Calcium, median (IQR)	9.50 (9.29–9.80)
Phosphorus, median (IQR)	3.42 (3.13–3.78)
Calciuria/24 h (mg/24 h), median (IQR)	144 (104–182)
Phosphaturia/24 h (mg/24 h), median (IQR)	621 (439–779)
PTH, median (IQR)	42.6 (35.4–52.4)
Vitamin D (nmol/L), median (IQR)	82.50 (68.75–102.50)
Bone assessment	
Bone densitometry (BMD)	
T-score L1-L4	
- Normal range (≥−1.0), n (%)	20 (33.33%)
- Osteopenia range (between −1.0 and −2.5), n (%)	23 (38.33%)
- Osteoporosis range (≤−2.5), n (%)	17 (28.33%)
T-score neck (femoral neck)	
- Normal range (≥−1.0), n (%)	19 (31.66%)
- Osteopenia range (between −1.0 and −2.5), n (%)	36 (60%)
- Osteoporosis range (≤−2.5), n (%)	5 (8.33%)

BMD, bone mineral density; LDL, low density lipoproteins; PTH, parathyroid hormone.

Osteoporosis (OP) (defined as a T-score ≤ −2.5 at the lumbar spine, femur, or femoral neck) was present in 18 patients (21% of the cohort), while osteopenia (T-score between −1 and −2.5) was observed in 33 patients (38%). Overall, 37 patients (42.5%) received supplementation with at least Vitamin D and/or calcium, and 13 patients (14.94%) were actively treated with amino bisphosphonates—specifically, 5 with alendronate, 1 with risedronate, 1 with neridronate, and 5 with zoledronic acid—or with teriparatide (1 patient). Twenty-two patients (25.29%) reported a family history of femoral or vertebral fractures, and 12 patients (13.79%) had a personal history of femoral or spinal fractures. Additionally, 37 patients (55%) were undergoing treatment with chondroprotective agents, including glucosamine sulfate, chondroitin sulfate, and/or hyaluronic acid, while 17 patients (19.5%) were on cyclical nonsteroidal anti-inflammatory drug (NSAID) therapy.

The CCI, calculated without age adjustment to estimate the burden of coexisting comorbidities, was generally low, with a maximum score of 5 out of 39 and an average score of 1.24 (±1.35).

### 3.1 Cardiovascular risk factors

#### 3.1.1 Diabetes

Only two patients had basal fasting glucose levels above 126 mg/dL, while 15 patients (17%) showed levels above 100 mg/dL at the time of screening. Additionally, six patients in the cohort were receiving treatment with insulin or metformin to manage their blood glucose levels. Importantly, these treated patients generally exhibited good glycemic control, with no markedly elevated glucose values that would suggest a significant diabetes-related impact. Furthermore, no correlations were found between fasting glucose levels among the 87 patients and any of the radiographic joint alterations evaluated.

#### 3.1.2 BMI and metabolic parameters

Twenty patients (23%) had a BMI between 25 and 30, while 8 patients (9%) were classified as obese (BMI >30). Regarding biomarkers, BMI showed a significant positive correlation with triglyceride levels (r = 0.48, p < 0.0001, 95% CI: 0.236–0.642) and a trend toward a negative correlation with HDL cholesterol (r = −0.27, p = 0.07, 95% CI: −0.494 to −0.029). No significant correlations were observed between BMI and total cholesterol, LDL cholesterol, or uric acid levels. However, a positive association was found between BMI and fasting glucose levels (r = 0.28, p = 0.02, 95% CI: 0.028–0.513). Regarding radiographic alterations*,* a moderate correlation was observed (r = 0.22, p = 0.04, 95% CI: 0.004–0.426) between BMI and joint space narrowing. However, no correlations were detected between BMI and AUSCAN or DREISER scores, nor with the number of swollen joints (SJ) or tender joints (TJ). Finally, no significant correlation was found between total cholesterol, triglyceride, and LDL levels, or hypertension, and the individual radiographic changes observed. However, a trend toward significance was observed between cholesterol levels and SJ (p = 0.20), while no correlation was found between SJ/TJ and the other recorded metabolic parameters.

### 3.2 Osteoporosis

When considered as a binary variable (0 = no OP, 1 = yes OP) based on T-scores value overall, OP showed no direct correlation with specific radiographic joint alterations. T-scores were assessed at the lumbar spine (L1–L4), femoral neck, and total femur, with analyses adjusted for age and disease duration.

Although not statistically significant, lower femoral neck T-scores were inversely associated with CRP levels, suggesting a potential link between bone loss and systemic inflammation. Lower femoral T-scores were significantly inversely correlated with AUSCAN scores (p = 0.0013, r = −0.4, 95% CI: −0.616 to −0.167) and DREISER scores (p = 0.02, r = −0.29, 95% CI: −0.521 to −0.029). Similarly, lower lumbar T-scores showed significant inverse associations with AUSCAN (p = 0.006, r = −0.36, 95% CI: −0.581 to −0.110) and DREISER scores (p = 0.02, r = −0.30, 95% CI: −0.528 to 0.033). Lower neck T-scores were also inversely correlated with AUSCAN scores (r = −0.37, p = 0.005, 95% CI: −0.582 to −0.110), with a trend toward significance observed for DREISER score (r = −0.25, p = 0.061, 95% CI: −0.488 to 0.020). These findings imply a negative impact of reduced bone mineral density (BMD) on hand function.

Conversely, no significant associations were found between T-scores, whether at the lumbar spine, femoral neck, or overall femur, and the number of tender joints (TJ), swollen joints (SJ), or VAS pain scores. However, the “worst” T-score recorded for each patient, regardless of the site measured, showed a trend toward significant negative correlation with joint malalignment (p = 0.08, r = −0.22, 95% CI: −0.462 to 0.038). Fracture risk, assessed using the FRAX algorithm, was available for 61 patients. In exploratory analyses, FRAX scores correlated significantly with several radiographic features of EHOA (Table 3). FRAX showed moderate positive associations with total osteophyte score (p < 0.0001, r = 0.48, 95% CI: 0.253–0.660), joint space narrowing (p = 0.0002, r = 0.47, 95% CI: 0.236–0.650), malalignment (p = 0.006, r = 0.35, 95% CI: 0.099–0.561), erosions (p = 0.002, r = 0.39, 95% CI: 0.145–0.592), and sclerosis (p = 0.001, r = 0.41, 95% CI: 0.163–0.604). No significant correlation was observed with the presence of cysts (p = 0.09, r = 0.22 95% CI: −0.039–0.458). Notably, these correlations were stronger than those found between radiographic alterations and lumbar or femoral T-scores, which only showed significant inverse associations with functional indices (AUSCAN and DREISER), but not with radiographic severity. This suggests that FRAX, by integrating BMD with clinical risk factors, captures more accurately the interplay between systemic bone fragility and local structural damage in EHOA.

**TABLE 3:** Correlation between FRAX score and radiographic features in erosive hand osteoarthritis (EHOA).

Radiographic feature	r (95% CI)	p-value
Osteophytes	0.482 (0.253–0.660)	<0.0001
Joint space narrowing	0.468 (0.236–0.650)	0.0002
Malalignment	0.351 (0.099–0.561)	0.0059
Erosions	0.391 (0.145–0.592)	0.0020
Sclerosis	0.407 (0.163–0.604)	0.0013
Cysts	0.224 (−0.039–0.458)	0.0851

### 3.3 Disease activity scores (AUSCAN, DREISER, VAS)

In exploratory analyses, AUSCAN and DREISER scores showed positive correlations with different radiographic changes, in particular subchondral cysts (AUSCAN: r = 0.22, p = 0.04, 95% CI: 0.009 to 0.422; DREISER: r = 0.30, p = 0.005, 95% CI: 0.092–0.488) and osteophytosis presence (AUSCAN: r = 0.23, p = 0.036, 95% CI: 0.014 to 0.426; DREISER: r = 0.23, p = 0.037, 95% CI: 0.013–0.425). However, no correlations were found between these scores and other joint radiographic alterations.

Additionally, AUSCAN and DREISER scores moderately positively correlated with the number of TJ (r = 0.53, p < 0.001, 95% CI: 0.353 to 0.675 and r = 0.44, p < 0.001, 95% CI: 0.251 to 0.609, respectively) and SJ (r = 0.26, p = 0.014, 95% CI: 0.047 to 0.463, and r = 0.29, p = 0.0069 95% CI: 0.078 to 0.487, respectively). Similarly, both the scores correlated moderately with the VAS pain scale (AUSCAN: r = 0.63, p < 0.001, 95% CI: 0.474–0.748; DREISER: r = 0.51, p < 0.001, 95% CI: 0.334–0.665), suggesting that increasing perceived pain corresponds to greater functional impairment and symptom’s severity. No correlations were observed between perceived pain and radiographic joint alterations except for osteophytosis.

Notably, perceived pain showed a moderate positive correlation with both the number of swollen joints (r = 0.42, p < 0.001, 95% CI: 0.223–0.588) and tender joints (r = 0.46, p < 0.001, 95% CI: 0.276–0.621).

### 3.4 Inflammatory parameters, CCI and disease duration

ESR showed a trend towards positive correlation with osteophytes (r = 0.31, p = 0.052, 95% CI: 0.091–0.508) and joint space narrowing (r = 0.29, p = 0.085, 95% CI: 0.072–0.494). CRP levels were positively correlated with osteophytosis (r = 0.71, p = 0.016, 95% CI: 0.137–1.301) but showed no association with other radiological parameters. Neither CRP nor ESR demonstrated correlations with AUSCAN or DREISER scores, VAS pain scores, or SJ and TJ counts. Finally, disease duration showed moderate positive correlations with radiographic scores for osteophytes (r = 0.64, p < 0.00, 95% CI: 0.494–0.756), joint space narrowing (r = 0.53, p < 0.01, 95% CI: 0.359–0.675), presence of erosions (r = 0.51, p < 0.01, 95% CI: 0.329–0.657), sclerosis (r = 0.47, p < 0.01, 95% CI: 0279 to 0.624), malalignment (r = 0.28, p = 0.0076, 95% CI: 0.072–0.476). No significant associations between the CCI and radiographic scores, nor with measures of disease activity, were observed.

## 4 Discussion

This is one of the largest monocentric retrospective cross-sectional cohort studies investigating the impact of cardiovascular and metabolic bone diseases on radiographic damage and disease activity indices in EHOA patients. Our findings suggest that selected comorbidities are associated with a higher symptom burden and greater radiographic severity in patients with EHOA. This aligns with a recently published review (Li et al., 2024) that examined various comorbidities, including those related to cardiovascular and metabolic systems, highlighting growing evidence that these conditions can have a substantial impact on osteoarthritis disease course.

Although the overall comorbidity burden—as measured by the CCI—did not appear to significantly affect structural joint damage or clinical disease activity in our cohort, some specific comorbidities were selectively associated with radiographic features of EHOA, as well as with clinical outcomes such as disability and pain.

Notably, both the AUSCAN score—which assesses pain, stiffness, and physical function—and the DREISER score—which assesses functional impairment—were significantly associated with tender (TJ) and swollen (SJ) joints count along with pain severity as measured by the VAS scale, which itself correlated with SJ/TJ count. These findings underscore the close interplay between pain and functional disability, highlighting their combined impact on patients’ quality of life.

Furthermore, AUSCAN and DREISER scores were associated with specific radiographic features, such as osteophytosis and subchondral cysts, suggesting that distinct bone alterations contribute to functional impairment. These associations reinforce the idea that skeletal changes in EHOA are not only structural markers of disease progression but also directly contribute to the symptomatic burden experienced by patients.

Regarding metabolic biomarkers, and in line with the findings by Brogren et al. (2024) who reported an association between elevated LDL cholesterol and greater OA severity, our study found that total serum cholesterol showed a trend toward correlation with the number of TJ, although it did not reach statistical significance. Nevertheless, this suggests a potential link between dyslipidemia-related signalling pathway alterations and increased joint pain sensitivity.

Only nine patients in our cohort were classified as obese (BMI >30), but 23% had a BMI >25. Obesity and metabolic syndrome are known to be strongly associated with OA, not only through mechanical stress that accelerates cartilage wear, osteophyte formation, and subchondral bone changes, but also through systemic inflammation. Adipose tissue indeed secretes adipokines (e.g., leptin, resistin) and cytokines (e.g., TNF-α, IL-6) which can perpetuate local inflammation and cartilage degradation (Batushansky et al., 2022). Several adipokine signaling pathways implicated in OA have been elucidated, encompassing both well-established pathways such as the AMP-Activated Protein Kinase/Mammalian Target of Rapamycin (AMPK/mTOR) pathway, the Nuclear Factor-κB (NF-κB) signaling pathway, and the Janus Kinase (JAK)/Signal Transducer and Activator of Transcription (STAT) pathway, as well as emerging pathways, including C/EBP-β and ATF4/RANKL (Zhang et al., 2022). These findings pave the way for novel therapeutic strategies that extend beyond the traditional focus on mitigating cartilage degradation. Additionally, the synovial adipokines may originate either from the secretion of the infrapatellar fat pad or from the bloodstream that permeates the synovial membrane and enters the joint cavity. Its strong association with OA has been extensively documented in the literature (Collins et al., 2020). Although hand joints are not weight-bearing joints, our study found a significant correlation between higher BMI and joint space narrowing in the hands. This cannot be explained by mechanical overload alone and instead suggests a metabolic-inflammatory mechanism linking BMI to structural joint damage. These findings align with the concept of EHOA as a systemic, metabolically influenced condition, reinforcing the role of obesity as a modifier of joint health, even in non-weight-bearing joints.

Moreover, diabetes is known to be associated with OA through hyperglycemia-mediated activation of molecular pathways involved in cartilage degradation and inflammation (Aiello et al., 2016). On the other hand, OA itself can contribute to systemic inflammation, with increased levels of cytokines such as TNF-α and IL-1, which in turn can worsen insulin resistance and facilitate diabetes onset. Recent findings by Marshall et al. (2019) suggest that diabetes may be a risk factor for OA progression, and previous studies have reported a higher pain perception in patients with both EHOA and diabetes (Magnusson et al., 2015).

In our cohort, the proportion of diabetic patients was small, with generally well-controlled disease, therefore, we are unable to draw strong conclusions regarding a potential association between diabetes and EHOA.

Overall, despite the long disease duration, our cohort consisted of patients who had access to high-quality care and were selected from a specialized tertiary care center. Therefore, these patients typically presented with milder comorbidity profiles, including the metabolic ones, which were effectively managed. This selection process means that the cohort likely represents a group of ‘healthier’ patients in terms of comorbidity burden, potentially leading to an overestimation of favorable outcomes. Secondly, while CCI is widely used, it captures only a limited range of comorbidities and does not account for their severity or duration. As a result, its utility for a comprehensive assessment of comorbidities in EHOA is constrained.

In terms of bone health, our findings corroborated previous studies highlighting an increased risk of OP in EHOA patients (Zoli et al., 2006; Tan et al., 2023; Mathieu et al., 2024) by emphasizing the role of bone fragility in worsening radiographic damage. Our study also revealed a novel association between FRAX-derived fracture risk and radiographic severity in EHOA. While T-scores at the femoral neck and lumbar spine correlated mainly with pain and functional indices (AUSCAN, DREISER), FRAX showed stronger and broader correlations with structural alterations such as osteophytes, joint space narrowing, erosions, and sclerosis. This discrepancy may be explained by the fact that FRAX incorporates not only bone density but also key clinical risk factors such as age, BMI, prior fractures, family history, smoking status, glucocorticoid exposure, and comorbidities (e.g., diabetes, rheumatoid arthritis). Additionally, patient selection bias may have influenced the final score. Therefore, FRAX may better reflect the cumulative systemic burden that contributes to bone fragility and joint degeneration.

Despite ongoing debate about the relationship between osteoporosis and OA, several studies, including one on over 200 EHOA patients (Mahajan et al., 2024) suggest a link between EHOA and increased fracture risk. Additionally, the concept of an ‘osteoporotic’ endotype of OA, characterized by bone fragility, has recently been proposed. This concept underscores that patients with erosive hand EHOA exhibit thinner bone mass and experience greater bone loss throughout the progression of the disease. Consequently, EHOA may be better conceptualized as a disorder of bone fragility, accompanied by an associated inflammatory burden, rather than simply a disease driven by cartilage degradation (McAlindon et al., 2021). Moreover, recent data from the QUALYOR cohort further supports that EHOA is a significant predictor of osteoporotic fractures in postmenopausal women, independent of traditional fracture risk factors (Auroux et al., 2025). From these findings we could speculate that the FRAX score could serve as an important prognostic tool for assessing EHOA damage and its progression.

These results support the hypothesis that EHOA is not merely a localised joint disease but part of a broader systemic metabolic bone phenotype, in which fracture risk and radiographic progression share common pathogenic pathways. These results should be interpreted as associative and hypothesis-generating. Future longitudinal studies are needed to clarify whether FRAX can predict radiographic progression over time, and whether targeting metabolic bone comorbidities can modify disease course.

The relationship between OA and OP remains a matter of debate, with some studies suggesting an inverse association (Foss and Byers, 1972) and others supporting a more direct link. Indeed, Güler-Yüksel et al. (2011) reported that EHOA progression over a 2-year period was associated with accelerated metacarpal BMD loss. Although we assessed BMD only in standard anatomical sites (lumbar spine, femur), our findings raise the question of whether targeted Dual-Energy X-ray Absorptiometry (DEXA) measurements at the hands might better capture local bone loss and its relationship with EHOA. In fact, Rovetta et al. (2003) found significantly higher serum levels of CTX-I, marker of bone resorption, in EHOA patients compared to those with non-erosive OA, suggesting that bone turnover may play a key role in EHOA pathogenesis. While CTX-I was not measured in our study, its inclusion may have provided invaluable insights into the interplay between cartilage degeneration and bone fragility. Further large-scale studies are needed to more precisely quantify the relationship between OP and erosive OA. Moreover, mechanistic research is essential to determine whether osteoporosis directly contributes to OA progression or merely shares common risk factors. Some studies have proposed that bisphosphonates, such as denosumab or clodronate, may have therapeutic potential in OA by modulating subchondral bone remodeling, reducing inflammation, and alleviating pain. However, robust clinical trials and larger studies are needed to confirm their efficacy and define clear indications in OA treatment.

Overall, the AUSCAN and DREISER scores effectively captured the burden of pain and functional disability in our cohort. These scores correlated directly with swollen and tender joints count, as well as perceived pain on the VAS scale, which in turn was associated with the occurrence of osteophytosis. These relationships suggest that analgesic therapy remains essential, alongside chondroprotective agents and joint support therapies, for controlling pain, particularly in patients with a longer disease duration—which also correlated with more severe radiographic changes in our patients.

We would remise not to mention some of the limitations of our study. Firstly, selection bias may affect the generalisability of our findings, as the cohort consisted of a population with access to a high level of care and milder comorbidity profiles. Secondly, although widely used, the CCI only captures a limited set of comorbidities and does not account for their severity or duration, thus limiting its utility for comprehensive comorbidity assessment in OA. Finally, although the relatively long disease duration in our cohort (mean 13.3 ± 8.8 years) allowed for the assessment of long-term structural damage, it also introduced confounders linked to concomitant comorbidities and varied treatment histories over time, thus complicating the attribution of cause-effect relationships. Given the exploratory retrospective design, no *a priori* sample size calculation was performed. Notably, the absence of longitudinal radiographic data limits our ability to assess the long-term progression of joint damage and bone loss in EHOA. Additionally, potential residual confounding due to treatment history could influence the results, as variations in pharmacological and non-pharmacological interventions may have impacted the disease course in some patients. Moreover, given the number of exploratory correlations performed and the absence of formal correction for multiple testing, the possibility of chance findings cannot be excluded, particularly for associations outside the main bone-related analyses.

Nevertheless, our findings suggest potential associations between metabolic and osteoporotic comorbidities and both symptom burden and radiographic severity in EHOA. These results support a multidisciplinary, personalised approach to EHOA management, emphasizing the need for early identification and management of relevant comorbidities. Further prospective, longitudinal studies are warranted to clarify these associations and guide more effective, patient-tailored therapeutic strategies.

## Data Availability

The raw data supporting the conclusions of this article will be made available by the authors, without undue reservation.
