# Supplementary material for: Impact of comorbidities in patients with erosive hand osteoarthritis (EHOA), a monocentric study
Source: Front Aging. 2026 Feb 12;7:1748066. doi: 10.3389/fragi.2026.1748066 (PMC12935983; doi:10.3389/fragi.2026.1748066)
Supplement: Supplementary file 1 [file Table1.docx]

**Supplementary Table 1S.** Clinical, laboratory and demographic data of the patients included

| Radiological Change | Joint type (n) | Grade | Findings in the examined joints, n (%) |
| --- | --- | --- | --- |
| **Osteophytes** | DIP (686) | grade 0; 1; 2; 3 | 99 (14,43%); 166 (24,20%); 211 (30,76%); 210 (30,61%) |
|  | PIP (688) | grade 0; 1; 2; 3 | 215 (31,25%); 207 (30,09%); 108 (15,70%); 158 (22,96%) |
|  | 1°IF (172) | grade 0; 1 | 51 (29,65%); 121 (70,35%) |
|  | TMC (172) | grade 0; 1; 2; 3 | 50 (29,07%); 58 (33,72%); 36 (20,93%); 28 (16,28%) |
|  | TS (172) | grade 0; 1 | 103 (59,88%); 69 (40,12%) |
|  | **DIP, PIP, TMC (1546)** | **grade 0; 1; 2; 3** | 364 (23,54%); 431 (27,88%); 355 (22,96%); 396 (25,61%) |
|  | 1°IF, TS (344) | grade 0; 1 | 154 (44,77%); 190 (55,23%) |
| **Joint space narrowing** | DIP (688) | grade 0; 1; 2; 3 | 32 (4,65%); 198 (28,78%); 193 (28,05%); 265 (38,52%) |
|  | PIP (688) | grade 0; 1; 2; 3 | 105 (15,26%); 299 (43,46%); 174 (25,29%); 110 (15,99%) |
|  | 1°IF (172) | grade 0; 1 | 71 (41,28%); 101 (58,72%) |
|  | TMC (172) | grade 0; 1; 2; 3 | 19 (11,05%); 48 (27,91%); 74 (43,02%); 31 (18,02%) |
|  | TS (172) | grade 0; 1 | 96 (55,81%); 76 (44,19%) |
|  | DIP, PIP, TMC (1548) | grade 0; 1; 2; 3 | 156 (10,08%); 545 (35,21%); 441 (28,49%); 406 (26,23%) |
|  | 1°IF, TS (344) | grade 0; 1 | 167 (48,55%); 177 (51,45%) |
| **Malalignment** | DIP (686) | grade 0; 1 | 404 (58,89%); 282 (41,11%) |
|  | PIP (688) | grade 0; 1 | 367 (53,34%); 321 (46,66%) |
|  | TMC (172) | grade 0; 1 | 87 (50,58%); 85 (49,42%) |
|  | DIP, PIP, TMC (1546) | grade 0; 1 | 858 (55,50%); 688 (44,50%) |
| **Erosions** | DIP (686) | grade 0; 1 | 308 (44,89%); 378 (55,10%) |
|  | PIP (688) | grade 0; 1 | 489 (71,07%); 199 (28,92%) |
|  | TMC (172) | grade 0; 1 | 123 (71,51%); 49 (28,49%) |
|  | DIP, PIP, TMC (1546) | grade 0; 1 | 920 (59,50%); 626 (40,49%) |
| **Sclerosis** | DIP (688) | grade 0; 1 | 335 (48,69%); 353 (51,31%) |
|  | PIP (688) | grade 0; 1 | 431 (62,64%); 257 (37,35%) |
|  | TMC (168) | grade 0; 1 | 70 (41,67%); 98 (58,33%) |
|  | DIP, PIP, TMC (1544) | grade 0; 1 | 836 (54,14%); 708 (45,85%) |
| **Cysts** | DIP (686) | grade 0; 1 | 370 (53,93%); 316 (46,06%) |
|  | PIP (688) | grade 0; 1 | 475 (69,04%); 213 (30,95%) |
|  | TMC (172) | grade 0; 1 | 136 (79,06%); 36 (20,93%) |
|  | DIP, PIP, TMC (1546) | grade 0; 1 | 981 (63,45%); 565 (36,54%) |

*Legend: DIP: Distal Interphalangeal; IF: Interphalangeal; PIP: Proximal Interphalangeal; TMC: Trapeziometacarpal; TS: trapezium-scaphoid;*
